# miR-337-3p and Its Targets *STAT3* and *RAP1A* Modulate Taxane Sensitivity in Non-Small Cell Lung Cancers

**DOI:** 10.1371/journal.pone.0039167

**Published:** 2012-06-18

**Authors:** Liqin Du, Maria C. Subauste, Christopher DeSevo, Zhenze Zhao, Michael Baker, Robert Borkowski, Jeoffrey J. Schageman, Rachel Greer, Chin-Rang Yang, Milind Suraokar, Ignacio I. Wistuba, Adi F. Gazdar, John D. Minna, Alexander Pertsemlidis

**Affiliations:** 1 Greehey Children's Cancer Research Institute, UT Health Science Center at San Antonio, San Antonio, Texas, United States of America; 2 Department of Cellular and Structural Biology, UT Health Science Center at San Antonio, San Antonio, Texas, United States of America; 3 Department of Pediatrics, UT Health Science Center at San Antonio, San Antonio, Texas, United States of America; 4 Division of Basic Sciences, Southwestern Graduate School of Biomedical Sciences, UT Southwestern Medical Center, Dallas, Texas, United States of America; 5 McDermott Center for Human Growth and Development, UT Southwestern Medical Center, Dallas, Texas, United States of America; 6 Simmons Comprehensive Cancer Center, UT Southwestern Medical Center, Dallas, Texas, United States of America; 7 Hamon Center for Therapeutic Oncology Research, UT Southwestern Medical Center, Dallas, Texas, United States of America; 8 Department of Pharmacology, UT Southwestern Medical Center, Dallas, Texas, United States of America; 9 Department of Internal Medicine, UT Southwestern Medical Center, Dallas, Texas, United States of America; 10 Department of Pathology, UT Southwestern Medical Center, Dallas, Texas, United States of America; 11 Department of Pathology, UT MD Anderson Cancer Center, Houston, Texas, United States of America; 12 Department of Thoracic/Head and Neck Medical Oncology, UT MD Anderson Cancer Center, Houston, Texas, United States of America; Virginia Commonwealth University, United States of America

## Abstract

NSCLC (non-small cell lung cancer) often exhibits resistance to paclitaxel treatment. Identifying the elements regulating paclitaxel response will advance efforts to overcome such resistance in NSCLC therapy. Using *in vitro* approaches, we demonstrated that over-expression of the microRNA miR-337-3p sensitizes NCI-H1155 cells to paclitaxel, and that miR-337-3p mimic has a general effect on paclitaxel response in NSCLC cell lines, which may provide a novel adjuvant strategy to paclitaxel in the treatment of lung cancer. By combining *in vitro* and *in silico* approaches, we identified *STAT3* and *RAP1A* as direct targets that mediate the effect of miR-337-3p on paclitaxel sensitivity. Further investigation showed that miR-337-3p mimic also sensitizes cells to docetaxel, another member of the taxane family, and that STAT3 levels are significantly correlated with taxane resistance in lung cancer cell lines, suggesting that endogenous STAT3 expression is a determinant of intrinsic taxane resistance in lung cancer. The identification of a miR-337-3p as a modulator of cellular response to taxanes, and *STAT3* and *RAP1A* as regulatory targets which mediate that response, defines a novel regulatory pathway modulating paclitaxel sensitivity in lung cancer cells, which may provide novel adjuvant strategies along with paclitaxel in the treatment of lung cancer and may also provide biomarkers for predicting paclitaxel response in NSCLC.

## Introduction

Paclitaxel is a microtubule-targeting agent initially isolated from the conifer *Taxus brevifolia* – the yew tree has a long history of medicinal uses [1] – and is widely used in the treatment of human cancers, including lung cancer. For NSCLC, resistance to paclitaxel is common, with response rates ranging from 21% to 24% [2], [3]. Mechanisms for such resistance include over-expression of P-glycoprotein, alterations in tubulin composition, and mutations in β-tubulin [4], [5], [6], [7]. A recent study indicated that a large group of protein-coding genes belonging to a wide range of functional classes is potentially involved in modulating paclitaxel resistance in cancer treatment [8]. Identifying the mechanisms regulating the expression of key genes involved in paclitaxel resistance will advance efforts to overcome such resistance in the treatment of lung cancer.

We are interested in the potential involvement of microRNAs (miRNAs) in modulating paclitaxel response in lung cancer treatment. miRNAs are short, 19 to 23 nucleotide RNAs found in multiple organisms that regulate gene expression largely by decreasing levels of target messenger RNAs [9], [10] and have been shown to play important roles in regulating a broad range of pathological processes, including cancer pathogenesis. miRNA levels can be easily manipulated using synthetic RNA molecules. A chemically stabilized, single-stranded RNA molecule complementary to a target miRNA acts as an inhibitor and decreases endogenous levels of the miRNA. Conversely, a double-stranded RNA molecule with one strand identical in sequence to a mature miRNA acts as a mimic of the naturally occurring miRNA and increases its cellular expression levels. Several studies have explored the therapeutic effects of miRNA mimics and inhibitors and demonstrated the potential of these classes of oligonucleotides as therapeutic agents [11], [12], [13], [14], [15], [16].

hsa-miR-337 (miR-337) is a human miRNA locus located at chromosome 14q32.2. miR-337-3p is highly expressed in normal immortalized fetal lung fibroblasts (IMR-90), and detectable in immortalized human bronchial epithelial cells (HBECs). The expression of miR-337-3p in lung cancer lines, however, is generally lower than in normal lung epithelial cell lines (**Figure S1**). Target prediction shows that miR-337-3p potentially regulates the expression of multiple genes that have been implicated in tumorigenesis. We found that miR-337-3p sensitizes lung cancer cells to paclitaxel treatment, but unexpectedly, does not significantly affect cell viability alone. We further used *in vitro* and *in silico* approaches to define the direct targets of miR-337-3p that mediate its effect on paclitaxel sensitivity. We also explored the potential relevance of miR-337-3p mimic and its targets in determining paclitaxel sensitivity in a large panel of NSCLC cell lines, and preliminarily explored the potential of miR-337-3p mimic as an adjuvant to paclitaxel treatment *in vitro* in NSCLC cell lines.

## Materials and Methods

### Cell lines

Cell lines used in this study were obtained from the Hamon Center for Therapeutic Oncology Research at UT Southwestern Medical Center. Lines beginning with “NCI-H" were established at the National Cancer Institute [17], [18]. Lines beginning with “HCC" and HBECs were established by the Hamon Center for Therapeutic Oncology Research at UT Southwestern Medical Center [19]. All lung cancer cell lines were grown in RPMI-1640 medium (Life Technologies, Carlsbad, CA) supplemented with 5% fetal bovine serum (Atlanta Biologicals, Lawrenceville, GA). HBECs were grown in GIBCO® KSFM medium supplemented with bovine pituitary extract and recombinant human epidermal growth factor (Life Technologies). All cell lines were DNA fingerprinted using the GenePrint PowerPlex 1.2 system (Promega, Madison, WI) and confirmed against fingerprint libraries maintained by ATCC and the Minna/Gazdar laboratory, and tested for contamination using the e-Myco Mycoplasma PCR detection Kit (Boca Scientific, Boca Raton, FL).

### Cell viability assays

Sensitivity of NSCLC cell lines to paclitaxel and docetaxel was measured using a standard protocol as described in Zhou et al. [20]. Briefly, cells were plated in 96-well format, with paclitaxel added in different concentrations after 24 h, followed by incubation with drug for 96 h. Cell viability was determined using the CellTiter 96® AQueous One Solution Cell Proliferation Assay (MTS, Promega). The effect of miR-337-3p and its targets were determined by transient transfection of either miR-337-3p mimic or siRNA oligos targeting specific genes. In general, cells were reverse-transfected with the indicated oligos in 96-well format and cultured for 72 h, followed by incubation with drugs for additional 72 h. Cell viability was determined using the CellTiter 96® AQueous One Solution Cell Proliferation Assay (MTS, Promega) or the CellTiter-Glo Luminescent Cell Viability Assay (ATP, Promega).

### Flow cytometry

Cells were transfected with the indicated oligos in 6-well plates for 48 h, followed by treatment with either paclitaxel or carrier for an additional 16 h. Both detached and attached cells were centrifuged at 1000 rpm for 5 min. The cells were washed once with 1X PBS (phosphate buffered saline) and fixed with 1X PBS containing 1 mM EDTA and 85% ethanol at 4°C. After 1 h, the cells were harvested by centrifugation at 1400 rpm for 5 min at 4°C. The cells were resuspended in 1X PBS, and treated with 50 µg/ml propidium iodide and 100 µg/ml RNase A for 30 min at 37°C. Cell cycle data were collected on a Cytomics FC 500 flow cytometer (Beckman Coulter, Brea, CA), with 20,000 events collected per sample. Data were evaluated using the FlowJo data analysis software, version 9.1 (TreeStar, Ashland, OR).

### Quantitative RT-PCR (qRT-PCR)

miRNA expression was measured on an ABI PRISM 7900 Sequence Detection System using TaqMan® microRNA Assays (Life Technologies) with RNU19 RNA expression as an internal control for normalization of RNA loading. mRNA expression was measured using TaqMan® Gene Expression Assays with GAPDH mRNA expression as an internal control. Threshold cycle times (C_t_) were obtained and relative gene expression was calculated using the comparative cycle time method.

### Western blots

Cell lysates were prepared using NP-40 buffer. Protein concentration was determined using the Pierce BCA assay (Thermo Fisher, Rockford, IL). For electrophoresis, equal amounts of cell lysate were resolved by SDS-PAGE and transferred to Immun-Blot PVDF membranes (Bio-Rad, Hercules, CA). Membranes were blocked and probed with the following antibodies: rabbit anti-RAP1A or anti-STAT3 (Cell Signaling Technology, Beverly, MA), or goat anti-actin (Santa Cruz Biotechnology, Santa Cruz, CA). Bound antibodies were detected with secondary antibodies conjugated with horseradish peroxidase (HRP) (Santa Cruz Biotechnology) and visualized by enhanced chemiluminescent (ECL) substrate (Thermo Fisher). Relative changes in protein levels were quantified by densitometry using the Quantity One software (Bio-Rad).

### miRNA target prediction

To identify the regulatory targets of miR-337-3p, we used the miRmate method developed in our lab as described previously [21]. Briefly, the method rewards complete complementarity at positions 2–8 of the miRNA (the seed region), mismatches and insertions in the central bulge at positions 9–11 of the miRNA, some complementarity at the 3′ end, and specific sequence composition at positions 1 (A) and 9 (A or C) of the miRNA, according to the findings of Lewis et al. [22].

### Luciferase reporter assays

The segment of the wildtype (WT) 3′UTRs containing the predicted target sites of *RAP1A* (NM_002884) and *STAT3* (NM_003150) were cloned downstream of the luciferase cDNA in pMIR-REPORT (Ambion, Austin, TX), a vector that contains both luciferase and β-galactosidase cDNAs under the control of separate mammalian promoter/terminator systems. The mutant constructs (*RAP1A* MU and *STAT3* MU) with position 3 (the 2^nd^ nucleotide in the target seed sequence) changed from G to A and position 1 (immediately 3′ to the seed sequence) changed from A to U, were made using the QuikChange Site Directed Mutagenesis Kit (Stratagene, La Jolla, CA). Luciferase activity and β-galactosidase activity were measured using the Luciferase Assay System and β-galactosidase Assay System (Promega), respectively.

### mRNA expression profiling

NCI-H1155 cells were transfected with miR-337-3p mimic or negative control mimic. Total RNA was isolated using mirVana™ miRNA Isolation Kit (Ambion), labeled and hybridized to HumanWG-6 V3 Expression BeadChips (Illumina, San Diego, CA) using standard protocols. Slides were scanned on an Illumina BeadStation and signal intensities were summarized using BeadStudio v3.3 (Illumina). Background subtraction and quantile normalization were performed using the MBCB algorithm [23].

### Correlation of expression with 3′UTR motif content

Correlation between changes in gene expression induced by miR-337-3p over-expression and motif content of their 3′UTRs was analyzed using sylamer (http://www.ebi.ac.uk/enright/sylamer), which computes cumulative and hypergeometric p values associated with small word occurrences in a ranked seq of larger sequences, in this case 7-mer occurrences across the set of RefSeq 3′UTRs, and using linear regression as implemented in miReduce [24], [25]. For the latter, each motif contained in the 3′UTRs is considered to contribute linearly to the fold change profile, and miReduce iteratively calculates the motifs that contribute most. The regression coefficient for each motif can be positive or negative, depending on whether the interaction results in a suppression or activation of expression of the target gene. In the case of over-expressing a miRNA, we expect the transcripts containing motifs complementary to the microRNA seed sequence to be reduced in expression, so that the motif would have a negative regression coefficient.

### Reverse-Phase Protein Array (RPPA)

Protein lysates were prepared using hot lysis buffer (2% SDS; 0.06 M Tris-Cl, pH 6.8; 5% glycerol) with proteinase and phosphatase inhibitors (Sigma-Aldrich, St. Louis, MO; Santa Cruz Biotechnology) and β-mercaptoethanol freshly added. Lysates were denatured by boiling for 5 min with supernatants obtained by centrifuging at 13,000 rpm for 7 min at 4°C. Before arraying on slides, the protein lysates were filtered through 96-well filter plates with 25 μm pore membranes (Phenix Research Products, Candler, NC) to remove aggregates. Equal amounts of lysates were arrayed in triplicate on ONCYTE® AVID^TM^ nitrocellulose film slides (Grace Bio-Labs, Bend, Oregon) using a SpotArray^TM^24 Microarray printing system (PerkinElmer, Waltham, MA) under 55–60% humidity. For immuno-staining, the slides were first incubated at RT in ReBlot Plus Mild Solution (EMD Millipore, Billerica, MA) for less than 7 min to relax protein structure and then washed in TBS-T buffer then incubated in Pierce SEA BLOCK blocking buffer (Thermo Fisher), blocked with avidin and biotin (Dako, Glostrup, Denmark), and incubated with either STAT3 (EMD Millipore) or pSTAT3 (S727, Cell Signaling Technology) antibody at 4°C overnight. Slides were then washed and incubated with biotinylated secondary antibodies (Vector Laboratories, Burlingame, CA) for 30 min followed by blotting with Qdot 655–streptavidin conjugate (Life Technologies) for 30 min. Slides were scanned with a ProScanArray Microarray Scanner (PerkinElmer). Protein expression levels were quantified using MicroVigene™ (Vigene Tech, Carlisle, MA) software and normalized for differences in protein loading using Sypro Ruby™ (Life Technologies) protein stain signals obtained on a separate slide printed in the same batch.

### miRNA expression profiling of tumor specimens

10–20 μm thick serial sections of 249 surgically resected NSCLC specimens (including 172 adenocarcinomas and 76 squamous cell carcinomas) were obtained using a Leica cryostat and homogenized using an Omni TH homogenizer (Omni International, Kennesaw, GA). Total RNA was isolated using TRI Reagent (Life Technologies) and quantified using a Nanodrop 1000 spectrophotometer (Thermo Fisher). RNA quality was determined on an Agilent 2100 Bioanalyzer (Agilent Technologies, Santa Clara, CA).

For microRNA analysis, the samples were labeled using the miRNA Complete Labeling and Hyb Kit and hybridized to Agilent Human miRNA microarray version 3 chips (Agilent Technologies), which contains probes for 866 human and 89 human viral microRNAs based on miRBase v12.0 (http://microrna.sanger.ac.uk). Hybridizations were performed in stainless steel SureHyb chambers (G2534A) at 55°C for 22 h, after which arrays were washed and scanned using an Agilent DNA Microarray Scanner (Agilent Technologies). miRNA expression levels were extracted using the Feature Extraction software (Agilent Technologies) and processed with the bioconductor [26] package AgiMicroRna to correct for background, remove control and un-detectable sequences, and quantile normalize and summarize the data.

## Results

### Over-expression of miR-337-3p sensitizes NCI-H1155 cells to paclitaxel and enhances paclitaxel-induced G_2_/M arrest

**Figure 1:**
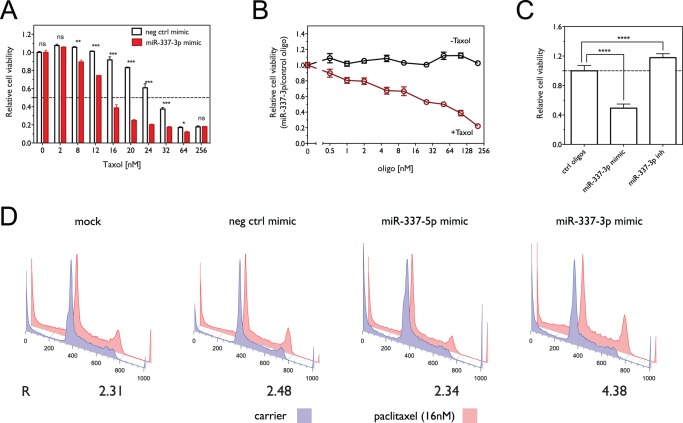
Over-expression of miR-337-3p sensitizes NCI-H1155 cells to paclitaxel. (A) Dose-dependent effect of paclitaxel on cell viability in the presence or absence of miR-337-3p mimic. Cell viability was measured using the MTS assay. (*, p<0.05; **, p<0.01; ***, p<0.001; ns, not significant) (B) Cell viability as a function of oligo concentration in the presence or absence of paclitaxel. Cell viability was measured using the ATP concentration assay. (C) Effect of miR-337-3p knockdown with miR-337-3p inhibitor (50 nM) on paclitaxel sensitivity in H1155 cells. Shown is the relative cell viability in the presence of 16 nM paclitaxel normalized to control oligos. (****, p<0.0001) (D) Cell cycle analysis as a function of miR-337-3p overexpression and paclitaxel treatment. The fraction of cells in G_1_, S and G_2_ phases was estimated using the Watson pragmatic model, with the ratio of G_2_ to G_1_ fractions for paclitaxel treated cells normalized to that observed for control conditions (R = [G_2_/G_1_]_paclitaxel_/[G_2_/G_1_]_carrier_).

To examine the effect of miR-337-3p on paclitaxel sensitivity in lung cancer cells, we used miR-337-3p mimic (Dharmacon) to increase miR-337-3p levels in NCI-H1155 cells, a lung cancer cell line characterized as moderately resistant to paclitaxel in a previous study [8]. As shown in **Figure 1A**, miR-337-3p over-expression significantly sensitizes cells to paclitaxel treatment, decreasing the IC_50_ – defined as the concentration that leads to a 50% decrease of cell viability – from 27.27 nM (95% CI 25.97–28.90) with control oligo to 14.60 nM (95% CI 14.08–15.15) with miR-337-3p mimic (p<0.0001). We next tested the dose-dependence of the effect of miR-337-3p on paclitaxel sensitivity and cell viability by transfecting NCI-H1155 cells with different concentrations of either miR-337-3p mimic or control oligo followed by treatment with either 16 nM paclitaxel or carrier. As shown in **Figure 1B**, miR-337-3p mimic significantly sensitizes cells to paclitaxel relative to the control oligo at concentrations as low as 0.5 nM (p = 0.0168), but does not significantly decrease cell viability in the absence of paclitaxel at concentrations even as high as 200 nM. To further evaluate the regulation of paclitaxel response by miR-337-3p, we examined the effect of miR-337-3p inactivation on paclitaxel sensitivity in H1155 cells. As shown in **Figure 1C**, inactivation of miR-337-3p by miR-337-3p inhibitor significantly decreases the response of H1155 to paclitaxel treatment, with relative cell viability increasing by 17.81±5.31% (p <0.0001), relative to control oligos.

Taxanes bind to the β subunit of tubulin and inhibit the normal dynamics of microtubule disassembly, leading to stabilized microtubules and prolonged arrest of cells in the G_2_/M phase of the cell cycle and eventually to cell death [27], [28]. We compared the effect of miR-337-3p over-expression on the cell cycle with or without paclitaxel treatment, at a time point (16 h) before paclitaxel induced significant apoptosis. As shown in **Figure 1D**, in the presence of paclitaxel, miR-337-3p mimic induces a dramatic G_2_/M arrest, with a normalized G_2_/G_1_ ratio of R = 4.38, relative to R = 2.48 for the control oligo, R = 2.34 for miR-337-5p mimic, and R = 2.31 for mock transfection, whereas miR-337-3p does not affect cell cycle distribution in the absence of paclitaxel.

The above results indicate that miR-337-3p modulates paclitaxel sensitivity in NCI-H1155 cells by specifically enhancing cell arrest in the G_2_/M phase of the cell cycle without significantly affecting cell survival alone.

### Expression array analysis indicates that many of the predicted targets of miR-337-3p are down-regulated at the mRNA level by miR-337-3p over-expression

**Figure 2:**
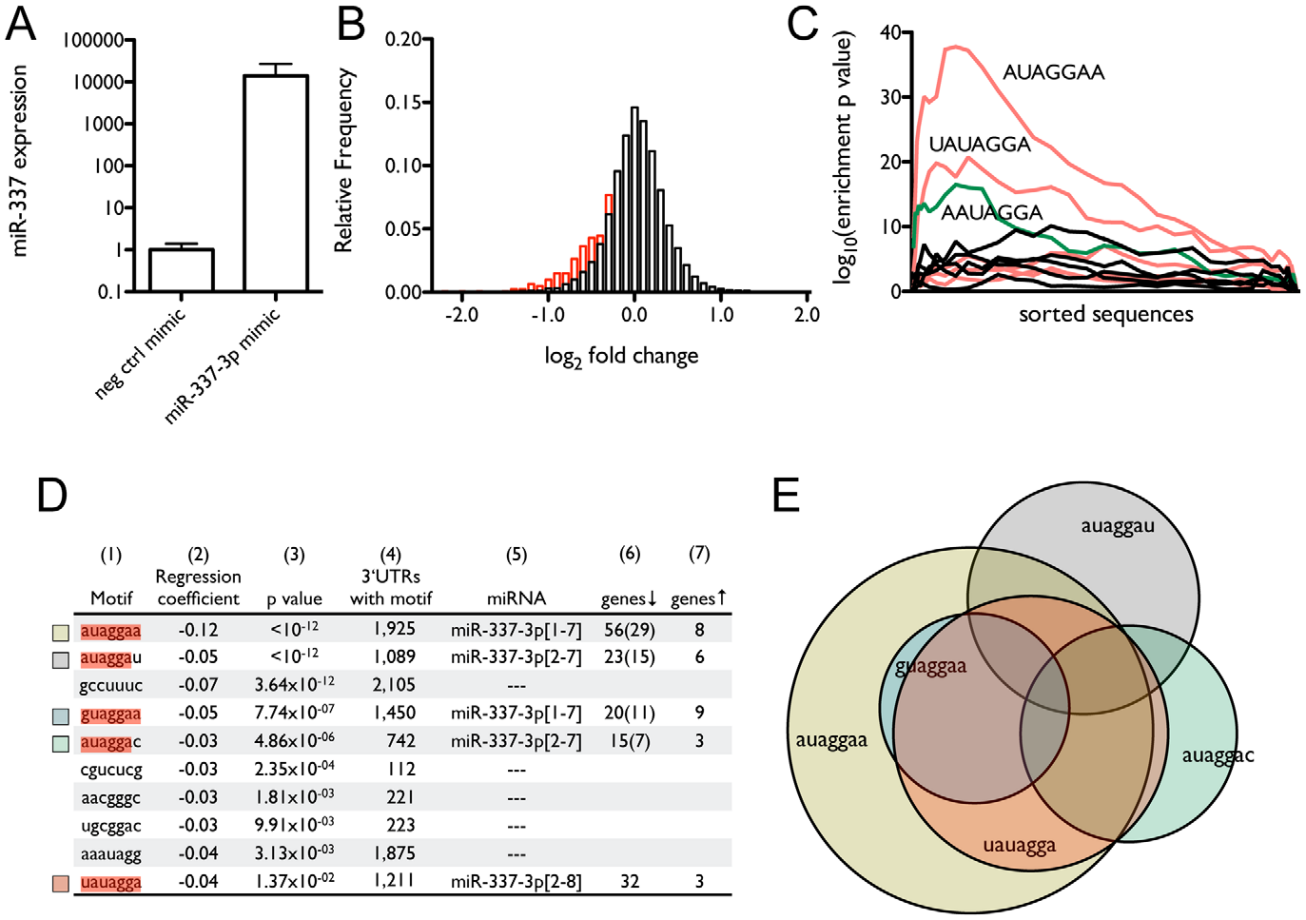
Predicted targets of miR-337-3p decrease in mRNA expression on over-expression of miR-337-3p. (A) Expression levels of miR-337-3p after 72 h transfection of NCI-H1155 cells with 50 nM miR-337-3p mimic as measured by qRT-PCR. (B) Histogram of changes in mRNA expression level as a function of miR-337-3p over-expression. The bars in black show relative frequencies of observed log_2_ fold changes for all genes, while the bars in red show the distribution for genes with 3′UTRs containing at least one 7-mer corresponding to the miR-337-3p seed region. (C) The significance landscape plot for 7-mers across all genes, sorted by change in expression, from most decreased to most increased, showing 7-mers that are highly enriched in the 3′UTRs of genes that are decreased in expression. (D) Correlation between mRNA expression and motif content of 3′UTRs induced by miR-337-3p over-expression. Shown are: (1) the 7-mer motif, highlighted to show complementarity to miR-337-3p, (2) the regression coefficient, (3) the p value, (4) the number of genes containing the motif, (5) the miRNA to which the motif corresponds, with the nucleotide positions fully complementary to the highlighted sequences in column 1 shown in brackets, and the number of genes that (6) decrease in expression or (7) increase in expression by ≥2-fold. In column 6, the number of genes that contain the listed motif, but do not contain the canonical miR-337-3p seed sequence target, is given in parentheses. (E) Proportional Venn diagram showing the relationship among the genes that decrease ≥2 fold in expression and contain one or more of the five 7-mers corresponding to the mature miR-337-3p sequences.

In order to comprehensively search for the target genes that mediate the effect of miR-337-3p on paclitaxel sensitivity of NCI-H1155 cells, we profiled gene expression by microarray to identify transcripts that are down-regulated by miR-337-3p over-expression, since increasing evidence shows that miRNAs regulate target gene expression through decreasing mRNA levels [29]. The magnitude of miR-337-3p over-expression (**Figure 2A**) is comparable to the differences in endogenous expression observed in different tissues and between IMR-90 fetal lung fibroblasts and HBECs (**Figure S1**). Since most recent studies support perfect complementarity between 7-mers in the seed region (covering positions 1–8) of a mature miRNA with sequences in the 3′UTR of mRNA transcript as a critical determinant of the interaction between a miRNA and a target gene [30], [31], we assessed the correlation between expression and motif content of mRNA transcripts based on the occurrence of 7-mers in their 3′UTRs. As shown in **Figure 2B**, significantly more of the 1,211 genes containing the 7-mer UAUAGGA complementary to the miR-337-3p seed sequence – bases 2–8, considered to be the strongest determinant of miRNA:target interaction – decrease in expression than increase (p = 1.37×10^−2^), among which 32 genes decrease ≥2 fold and only 3 genes increase ≥2 fold (**Figure 2D**). In contrast, roughly equal numbers of the 19,262 genes not containing the 7-mer decrease and increase in expression. Furthermore, **Figures 2C and 2D** show that among the 16,384 7-mer motifs occurring in the 3′UTRs of human mRNA transcripts, several are over-represented in the 3′UTRs of genes that decreased in cells transiently transfected with miR-337-3p mimic relative to a negative control mimic (**Figure 2C**), with only 10 motifs showing a significant negative correlation between mRNA expression levels and their presence in 3′UTRs (**Figure 2D**). 5 of the motifs correspond to the seed region of a known miRNA (miR-337-3p), including 1 motif corresponding to bases 2–8 and 4 corresponding to positions 1–7 and 2–7, consistent with recent work on seed region matches [30], [31]. Interestingly, we found that one motif (GUAGGAA) contains a G:U basepair at position 7, suggesting that the determinants of miRNA targets may include non-Watson:Crick complementarity. **Figure 2E** shows that, although many of the target genes identified by the 5 motifs overlap, the additional 4 motifs identify unique targets. Overall, the above results show that a significant number of genes containing putative miR-337-3p target sites are down-regulated by miR-337-3p at the mRNA level and highlight the value of a comprehensive search for miRNA targets based on 7-mer complementarity to the seed region.

### *STAT3* and *RAP1A* are the direct targets that mediate the effect of miR-337-3p on paclitaxel sensitivity

**Figure 3:**
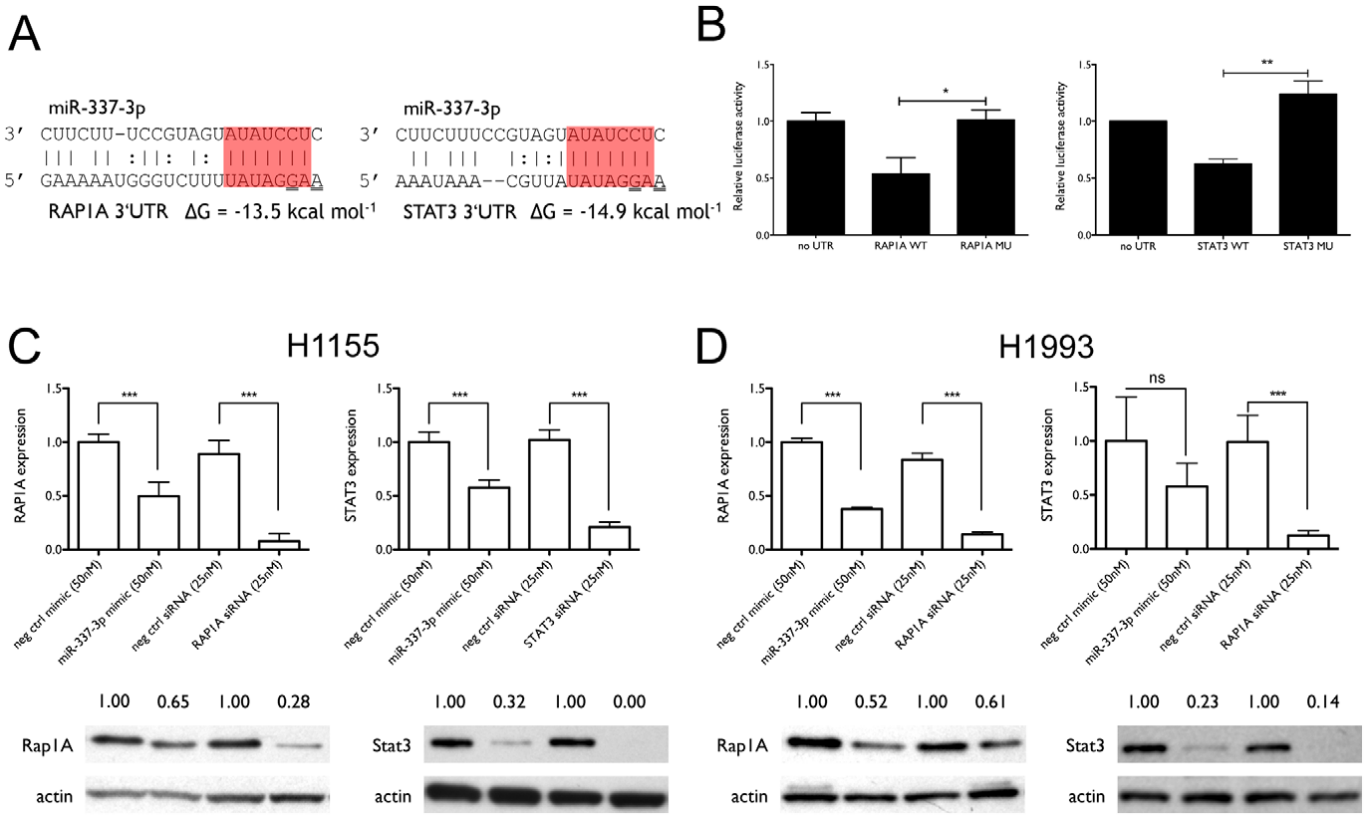
*RAP1A* and *STAT3* are direct targets of miR-337-3p. (A) miR-337-3p and its predicted interaction with target sites in *RAP1A* and *STAT3*. Shown are the structures and sequences of the miRNA:target interactions for miR-337-3p and the 3′UTRs of *RAP1A* and *STAT3*, and the predicted free energy of hybridization. The seed sequence is highlighted in red. Bases altered by site-directed mutagenesis are underlined. (B) Luciferase reporter assay. NCI-H1155 cells were co-transfected with the indicated oligos and the luciferase reporter vectors. Luciferase and β-galactosidase activities were measured after 72 h, with luciferase activity normalized to β-galactosidase activity. (C) Expression of *RAP1A* and *STAT3* mRNA and protein levels after 72 h of transfection of NCI-H1155 cells with either 50 nM miR-337-3p, 25 nM siRNA directed against each of the genes or negative control oligos. Shown are average qRT-PCR results of three independent transfections and representative Western blot images and quantification of band intensities. (D) *RAP1A* and *STAT3* mRNA and protein expression in NCI-H1993 cells. *, p<0.05; **, p<0.01; ***, p<0.001.

Among the genes down-regulated by miR-337-3p, *RAP1A* and *STAT3*, which are down-regulated by 60% and 63% by miR-337-3p over-expression, respectively, are both predicted to be direct targets of miR-337-3p (**Figure 3A**) and are potentially involved in the regulation of paclitaxel sensitivity. To validate these interactions, we constructed luciferase reporter vectors containing the wildtype 3′UTRs of *STAT3* and *RAP1A* and the corresponding control 3′UTRs with mutations in the first and third nucleotides of the target site sequences (**Figure 3A**), which have been shown to be crucial in disrupting miRNA:target interactions [22]. As shown in **Figure 3B**, miR-337-3p over-expression significantly decreased luciferase activity in cells expressing the wildtype 3′UTRs as compared with no 3′UTR or mutated 3′UTRs, demonstrating that miR-337-3p interacts directly with specific sites in the 3′UTRs of *STAT3* and *RAP1A*. **Figure 3C** shows that the effect of miR-337-3p on mRNA expression levels of the two genes is coupled with down-regulation of the endogenous protein expression levels in H1155 cells. We further examined the effect of miR-337-3p overexpression on STAT3 and RAP1A levels in H1993, a NSCLC cell line that is defined as paclitaxel-resistant, with an undefined IC_50_. As with H1155 cells, miR-337-3p also down-regulates STAT3 and RAP1A levels at mRNA and protein levels in H1993 cells, as shown in **Figure 3D**. These results suggest that miR-337-3p has a general regulatory effect on STAT3 and RAP1A expression in lung cancer cells.

**Figure 4:**
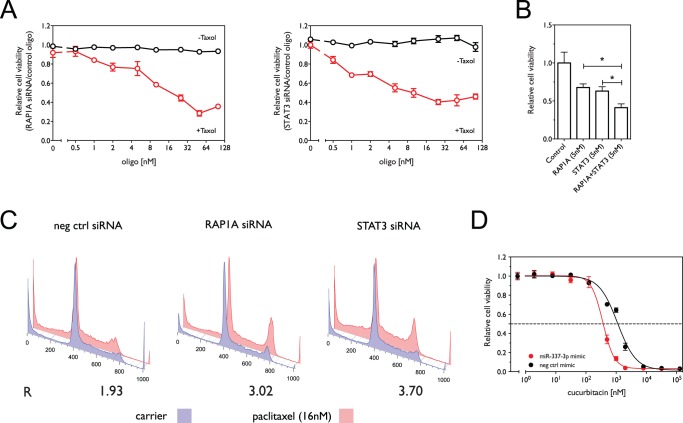
siRNA knockdown of regulatory targets of miR-337-3p sensitizes NCI-H1155 cells to paclitaxel. (A) Effect of knockdown of *RAP1A* and *STAT3* expression by siRNA on cell viability in the presence and absence of paclitaxel. Shown in red is cell viability in 16 nM paclitaxel normalized to viability in carrier as a function of increasing concentrations of siRNAs against *RAP1A* or *STAT3*. Shown in black is cell viability in the absence of paclitaxel. (B) Effect of combined knockdown of *RAP1A* and *STAT3* on paclitaxel sensitivity. Shown is the relative cell viability in the presence 16 nM paclitaxel normalized to control oligos. (C) Knockdown of STAT3 and RAP1A enhances paclitaxel-induced G_2_/M arrest as measured by flow cytometry. The ratio (R) of the G_2_ to G_1_ fractions induced by paclitaxel treatment was determined as above. (D) Cell viability as a function of oligo concentration (miR-337-3p mimic or negative control mimic) in the presence of cucurbitacin. *, p<0.05.

In order to evaluate whether *STAT3* and *RAP1A* mediate the effect of miR-337-3p on paclitaxel sensitivity, we knocked down each gene individually and both of them together. **Figure 4A** shows that knockdown of *STAT3* and *RAP1A* increased the sensitivity of NCI-H1155 cells to paclitaxel treatment as compared with control oligos, but did not significantly affect cell survival in the absence of paclitaxel. Combining the two siRNAs at half the concentration leads to a greater decrease in cell viability than observed with either siRNA alone (p = 0.017 for *RAP1A* and p = 0.041 for *STAT3*), which suggests that the combined knockdown has a synergistic effect on paclitaxel response (**Figure 4B**). Moreover, **Figure 4C** shows that knockdown of *STAT3* or *RAP1A* significantly enhances the G_2_/M arrest under paclitaxel treatment as compared with control oligos, with normalized G_2_/G_1_ ratios of R = 3.02 and R = 3.70, respectively, relative to R = 1.93 for the controls and comparable to R = 4.37 for transfection with the miR-337-3p mimic.

Increasing evidence supports the application of STAT3 inhibitors, such as cucurbitacin, as therapeutic agents in treating various cancers. We therefore tested the effect of miR-337-3p over-expression on the response of NCI-H1155 cells to treatment with cucurbitacin, the cytotoxicity of which is at least partially based on the inhibition of STAT3 activity [32], [33]. As shown in **Figure 4D**, miR-337-3p mimic sensitizes cells to cucurbitacin, decreasing the IC_50_ from 1.14 μM (95% CI 1.03–1.28) to 0.37 μM (95% CI 0.33–0.42) (p <0.0001), indicating that targeting both *STAT3* expression with miR-337-3p mimic and STAT3 activation with cucurbitacin has a synergistic effect on cancer cell survival, and suggesting that miR-337-3p may also serve as an adjuvant to STAT3 inhibitors.

### miR-337-3p sensitizes both paclitaxel-sensitive and paclitaxel–resistant cell lines as well as those from different histological subtypes

**Figure 5:**
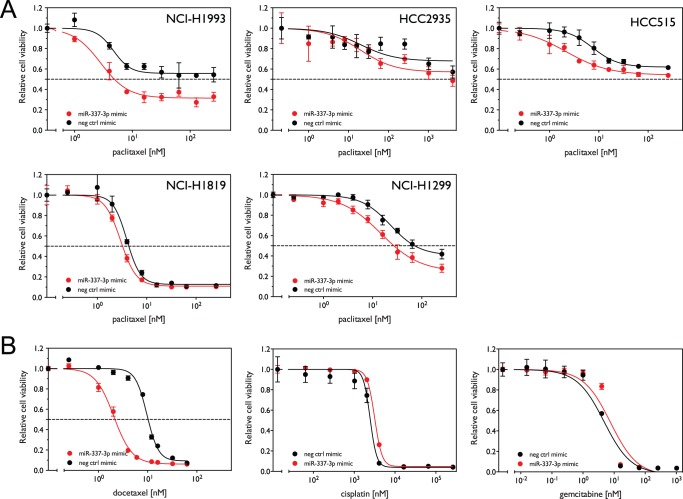
miR-337-3p over-expression selectively sensitizes cells to drugs that affect microtubule dynamics. (A) Five cell lines were transfected with 50 nM miR-337-3p mimic or negative control mimic oligo for 72 h. Cells were then treated with increasing concentrations of paclitaxel for an additional 72 h. Relative cell viability was measured and calculated as above. (B) NCI-H1155 cells were treated with 50 nM miR-337-3p mimic in the presence of increasing concentrations of docetaxel, cisplatin and gemcitabine as described above. miR-337-3p mimic sensitizes calls to docetaxel relative to control oligos, shifting the dose-response curve to the left, but does not sensitize cells to cisplatin and gemcitabine.

In order to test the generality of the effect of miR-337-3p on paclitaxel sensitivity in lung cancer cells, we selected five additional cell lines belonging to different histological subtypes and with distinct responses to paclitaxel (**Table S1**). As shown in **Figure 5A**, miR-337-3p over-expression sensitized all five cell lines to paclitaxel treatment, with a larger effect in the cell lines that are relatively resistant to paclitaxel treatment (upper panels) as compared to cell lines that are relatively sensitive (lower panels).

### miR-337-3p selectively sensitizes cells to taxanes

In order to examine the specificity of miR-337-3p in modulating sensitivity to paclitaxel, we tested the effect of miR-337-3p over-expression on the sensitivity of H1155 cells to other chemotherapeutic agents that are commonly used in the treatment of lung cancer. As shown in **Figure 5B**, transient transfection with miR-337-3p mimic sensitizes NCI-H1155 cells to docetaxel, another member of the family of taxanes, leading to a greater sensitization than that observed with paclitaxel (IC_50_ of 10.22 nM with a 95% CI 9.68–10.78 for the control oligo and 2.16 nM with a 95% CI 2.02–2.32 for miR-337-3p mimic, p <0.0001). **Figure 5B** also shows that miR-337-3p over-expression does not sensitize cells to treatment with cisplatin, a platinum-based chemotherapy agent which induces cell death by cross-linking subunits of DNA (IC_50_ of 2.49 μM with a 95% CI 2.22–3.09 for the control oligo to 3.2 μM with a 95% CI 3.12–3.35 for miR-337-3p mimic), and slightly de-sensitizes them to treatment with gemcitabine, a nucleoside analog which arrests cell growth by disrupting the normal DNA replication and repair (IC_50_ of 4.59 nM with a 95% CI 3.32–6.31 for the control oligo to 7.16 nM with a 95% CI 5.22–9.77 for miR-337-3p mimic, p = 0.04). These findings demonstrate the specificity of miR-337-3p in enhancing the cytotoxicity of microtubule stabilizing agents, further suggesting that a major role of miR-337-3p is to regulate microtubule dynamics during mitosis in lung cancer cells.

### Correlation analysis in a panel of NSCLC lines suggests that STAT3 may be the major endogenous determinant of intrinsic paclitaxel response

**Figure 6:**
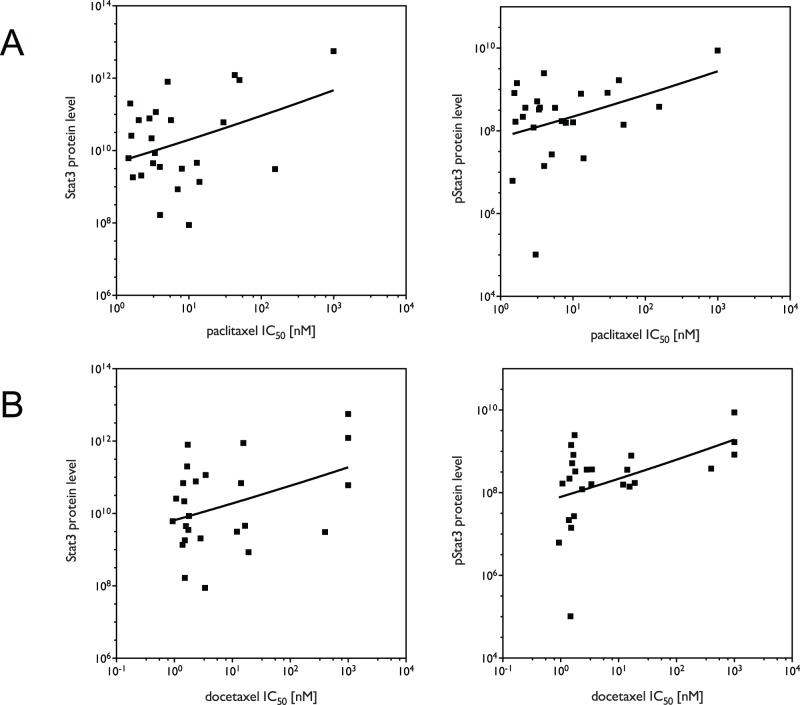
STAT3 and pSTAT3 levels correlate with resistance to taxanes in a panel of NSCLC cell lines. STAT3 and pSTAT3 protein levels were measured by RPPA. IC_50_s were measured by MTS assay. Protein levels plotted against IC_50_s for (A) paclitaxel and (B) docetaxel in 25 NSCLC cell lines. Correlations assumed that data were sampled from Gaussian populations (Pearson), and significance was assessed by one-tailed test.

The involvement of STAT3 in tumorigenesis is well recognized and therapeutic agents specifically inhibiting STAT3 signaling have been used to treat several types of cancers [34]. However, the role of STAT3 in lung cancer tumorigenesis and therapy has not been fully addressed. We therefore investigated the relevance of STAT3 to the sensitization of lung cancer cells to paclitaxel treatment, by assessing the correlation between paclitaxel response, protein levels of total STAT3 and phospho-STAT3 (pSTAT3) and levels of miR-337-3p in a panel of NSCLC cell lines. As shown in **Figure 6A and B**, both total STAT3 and pSTAT3 protein levels are significantly correlated with both paclitaxel (r = 0.44, p = 0.013 for STAT3 and r = 0.37, p = 0.035 for pSTAT3) and docetaxel (r = 0.48, p = 0.008 for STAT3 and r = 0.043, p = 0.017 for pSTAT3) response across the panel of cell lines. We also attempted to assess the correlation between miR-337-3p expression levels, taxane response and levels of STAT3 and pSTAT3, but miR-337-3p was not detectable in most of the lung cancer cell lines, and therefore not amenable to such an analysis (data not shown). Overall, the above results suggest that the endogenous expression of STAT3 plays an important role in determining the intrinsic sensitivity of lung cancer cells to taxane treatment, and that the expression levels of STAT3 may serve as a biomarker for predicting taxane response in NSCLC.

### Tumor levels of STAT3 and miR-337-3p are correlated with overall survival in NSCLC patients

**Figure 7:**
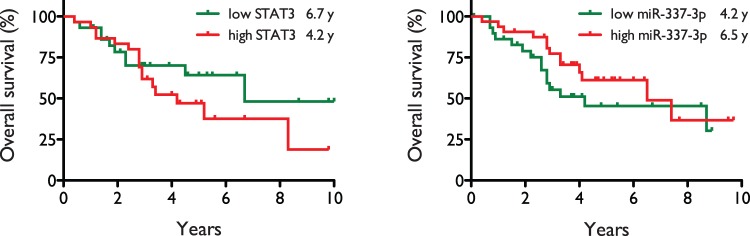
Overall survival of NSCLC patients correlates with tumor levels of STAT3 and miR-337-3p. Shown are survival curves for groups of NSCLC patients with the lowest and highest tumor expression levels of STAT3 and miR-337-3p. Mean survival times in the low (n_L_ = 29, green) and high STAT3 (n_H_ = 30, red) groups were 6.7 years and 4.2 years, respectively, with a hazard ratio of 1.54 (95% CI 0.71–3.35), and a p value of 0.28, while mean survival times in the low (n_L_ = 30, green) and high miR-337-3p (n_H_ = 32, red) groups were 4.2 years and 6.5 years, respectively, with a hazard ratio of 0.65 (95% CI 0.30–1.38) and a p value of 0.26. Statistical significance was assessed by Mantel-Cox log-rank test.

Shown in **Figure 7** are survival curves for the patients with the lowest and highest tumor expression levels of STAT3 and miR-337-3p, as measured by Illumina and Agilent microarray, respectively. Mean survival in the low and high STAT3 groups (n_L_ = 29, n_H_ = 30) were 6.7 years and 4.2 years, respectively, with a hazard ratio of 1.54 (95% CI 0.71–3.35), and a p value of 0.28, assessed by Mantel-Cox log-rank test. Mean survival in the low and high miR-337-3p groups (n_L_ = 30; n_H_ = 32) were 4.2 years and 6.5 years, respectively, with a hazard ratio of 0.65 (95% CI 0.30–1.38) and a p value of 0.26, assessed as above. Although the results did not reach statistical significance by Mantel-Cox test, there is a clear trend indicating that high miR-337-3p levels and low STAT3 levels are correlated with increased overall survival.

## Discussion

In this study, we define a novel pathway by which cellular sensitivity to paclitaxel is regulated by miR-337-3p and its two direct regulatory targets, *STAT3* and *RAP1A*. We have shown that miR-337-3p sensitizes lung cancer cell lines to paclitaxel treatment by directly down-regulating the expression of *STAT3* and *RAP1A*, leading to increased cell death by enhancing the paclitaxel-induced arrest of cells in the G_2_/M phase of the cell cycle. In addition, we are the first to define the regulation of *STAT3* and *RAP1A* by a single miRNA in the context of lung cancer, and provide the first preliminary evidence showing that STAT3 expression is an intrinsic determinant of paclitaxel response in lung cancer cells. Given the increasing evidence supporting clinical application of STAT3 inhibitors as therapeutic agents in treating various cancers [32], [33], our findings underscore the relevance of targeting *STAT3* in lung cancer therapy from at least two perspectives. First, our findings provide a potential alternative, the use of miR-337-3p mimic, to target *STAT3*. Second, our findings provide support for combining STAT3 inhibitors with paclitaxel in order to improve response to lung cancer treatment.

Previous studies indicate that miRNAs act as fine-tuning regulators of protein expression. As we observed in this study, miR-337-3p decreases the mRNA and protein levels of STAT3 and RAP1A less than specific siRNA oligos. However, the fact that one miRNA can disrupt multiple pathways that are involved in regulating cancer cell survival or drug response in the same direction both minimizes the chances of off-target effects and decreases the possibility of acquired resistance to miRNA-based therapeutic strategies relative to traditional therapeutic approaches, which may therefore increase the proportion of cancer patients that will respond to such treatments and the duration of that response. This is especially important given the fact that individual therapeutic agents currently used exhibit resistance rates ranging from 50%-80% [35]. Our examination suggests that miR-337-3p sensitizes lung cancer cells with different tumorigenic and molecular backgrounds, supporting the potential of miR-337-3p mimic as a relatively universal therapeutic adjuvant to paclitaxel in the treatment of lung cancer.

STAT3 is a well-characterized transcription factor that has been demonstrated to contribute to various processes of tumorigenesis, such as tumor cell survival and proliferation, invasion, angiogenesis and drug resistance [36]. Many of its target genes, such as cyclin D1, c-myc, Bcl-2, survivin and p21^cip1^ have been shown to control tumor cell survival and proliferation [37], [38]. However, the mechanisms by which STAT3 regulates drug resistance have not been clearly defined. Hawthorne *et al*., showed that STAT3 activation induced subsequent up-regulation of p21^cip1^ and paclitaxel resistance in breast cancer [39]. Real *et al*., showed that treatment of a metastatic breast cancer cell line with a dominant-negative form of STAT3 sensitized cells to paclitaxel and inhibited STAT3-mediated Bcl-2 induction [40]. These studies, however, did not demonstrate direct involvement of either p21^cip1^ or Bcl-2 in mediating STAT3-induced paclitaxel resistance. Other studies have demonstrated pathways by which STAT3 modulates microtubule dynamics independent of its function as a transcription factor. For example, Ng, *et al.* showed that STAT3 regulates cell migration in murine embryonic fibroblast (MEF) cells by directly interacting with stathmin [41], a protein that binds the α/β-tubulin heterodimers to facilitate the depolymerization of microtubules. This study also shows that the interaction of STAT3 with stathmin does not significantly affect the cell cycle distribution, which is consistent with our observations showing that *STAT3* knockdown alone does not affect cell cycle distribution. A more recent study showed the physical association of pSTAT3 with tubulin, suggesting another possible mechanism of STAT3 modulation of paclitaxel sensitivity [42].

RAP1A is one of the two isoforms of RAP1 and has also been implicated in regulating microtubule dynamics. RAP1 has been indicated to activate the MAPK/ERK pathway [43], [44], [45], which has been implicated in regulating microtubule dynamics by phosphorylating microtubule-associated proteins MAP2 and MAP4 [46], [47]. RAP1 has also been shown to be important for interactions between cells and the extracellular matrix [48], and independent work has connected integrity of the extracellular matrix to sensitivity to paclitaxel [49].

**Figure 8:**
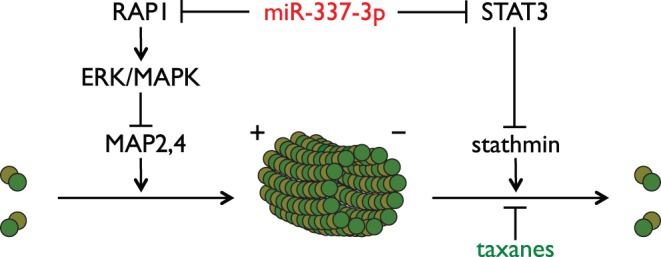
Proposed model of taxane sensitization mediated by miR-337-3p and its targets RAP1A and STAT3.

Based on the above evidence, we propose the model for the regulation of paclitaxel sensitivity by STAT3 and RAP1A shown in **Figure 8**. In this model, in normal microtubule dynamics, STAT3 acts as an antagonist of microtubule depolymerization by interacting with stathmin, and RAP1A inhibits microtubule polymerization through activation of ERK/MAPK and phosphorylation of MAP2 and MAP4. Knockdown of either *RAP1A* or *STAT3* alters the normal dynamics, making the cell vulnerable to perturbations caused by microtubule targeting agents. In the case of both taxane treatment and RAP1A or STAT3 depletion, the altered microtubule dynamics resulting from reduced RAP1A and STAT3 expression and the stabilization induced by taxanes have a synergistic effect on abrogating normal microtubule function, leading to enhanced G_2_/M arrest and eventually cell death. Further studies are clearly needed to validate the proposed model.

Another interesting observation in our study is that neither the over-expression of miR-337-3p nor the specific knockdown of *STAT3* and *RAP1A* significantly decrease cell viability or induce G_2_/M arrest alone, but rather enhance G_2_/M arrest and cell death only under conditions of paclitaxel treatment, even though its regulatory targets, *STAT3* and *RAP1A*, have been indicated to promote cancer cell survival and proliferation [50], [51]. This observation can be explained, at least partially, by the diversity of the STAT3 and RAP1A functions. The diverse and seemingly contradictory functions of STAT3 in different cellular contexts have been well documented in previous studies [36], [52], demonstrating that, although STAT3 targets multiple genes involved in tumorigenesis, not all of the target genes are expressed in a STAT3-dependent manner in all tumors. Rather, STAT3 controls distinct subsets of genes in different intracellular contexts. It is therefore possible that, in at least a subset of lung tumors, STAT3 primarily controls pathway(s) that specifically affect taxane sensitivity, but does not significantly affect the function of genes directly involved in controlling tumor cell survival. Evidence from our study supporting this possibility is that we did not observe down-regulation of p21^cip1^, a major target of STAT3 transcriptional activation, following either *STAT3* siRNA or miR-337-3p transfections (data not shown). This diversity of STAT3 functions in different tumor cellular contexts certainly warrants further investigation.

In summary, in this study we demonstrate for the first time the involvement of miR-337-3p in modulating drug resistance of lung cancer cells and define a novel regulatory pathway modulating paclitaxel response in lung cancer cells mediated by miR-337-3p down-regulation of *STAT3* and *RAP1A*, which has potential significance for the prediction and improvement of therapeutic response to taxane treatment in NSCLCs. Further work is clearly required to elucidate the genes down-stream of *STAT3* and *RAP1A* that mediate their effect on paclitaxel sensitivity and to define the feasibility and efficacy of miR-337-3p mimic delivery in the context of taxane-based therapies.

## Supporting Information

Figure S1 **Endogenous miR-337-3p expression levels in lung cell lines.** **s**miR-337-3p levels were measured by qRT-PCR. Shown are relative expression levels, normalized to levels observed in H1299, a NSCLC cell line that has the lowest endogenous level of miR-337-3p among the eight cell lines examined.
(EPS)

Table S1 **Cell lines tested for miR-337-3p-induced sensitization to paclitaxel.** NSCLC cell lines used in this study annotated as to tumor type, tumor subtype, age, ethnicity and gender of the patient from whom the line was derived, source, anatomical site, and IC_50_ to paclitaxel.
(DOC)
