# Supplementary material for: miR-337-3p and Its Targets STAT3 and RAP1A Modulate Taxane Sensitivity in Non-Small Cell Lung Cancers
Source: PLoS One. 2012 Jun 18;7(6):e39167. doi: 10.1371/journal.pone.0039167 (PMC3377607; doi:10.1371/journal.pone.0039167)
Supplement: Table S1 — Cell lines tested for miR-337-3p-induced sensitization to paclitaxel. NSCLC cell lines used in this study annotated as to tumor type, tumor subtype, age, ethnicity and gender of the patient from whom the line was derived, source, anatomical site, and IC50 to paclitaxel. (DOC) [file pone.0039167.s002.doc]

**Supplementary Material**

**Table S1.**

| **Cell Line** | **Tumor Type** | **Tumor Subtype** | **Age** | **Ethnicity** | **Gender** | **Tumor Source** | **Anatomical Site** | **IC_50_ (nM)** |
| --- | --- | --- | --- | --- | --- | --- | --- | --- |
| H1155 | Lung | Large Cell Neuroendocrine | 36 | Caucasian | M | metastasis | lymph node | 27 |
| H1299 | Lung | Large Cell Neuroendocrine | 43 | Caucasian | M | metastasis | lymph node | 65 |
| H1819 | Lung | Adenocarcinoma | 55 | Caucasian | F | metastasis | lymph node | 5 |
| H1993 | Lung | Adenocarcinoma | 47 | Caucasian | F | metastasis | lymph node | undefined |
| HCC2935 | Lung | Adenocarcinoma | 39 | Caucasian | M | primary | lung | undefined |
| HCC515 | Lung | Adenocarcinoma | 39 | Caucasian | F | metastasis | lymph node | undefined |
